# Supplementary material for: Health Emergency Disaster Risk Management of Public Transport Systems: A Population-Based Study after the 2017 Subway Fire in Hong Kong, China
Source: Int J Environ Res Public Health. 2019 Jan 15;16(2):228. doi: 10.3390/ijerph16020228 (PMC6351960; doi:10.3390/ijerph16020228)
Supplement: Supplementary file 1 [file ijerph-16-00228-s001.pdf]

Supplementary Material

Health Emergency Disaster Risk Management of Public Transport Systems: A Population-based Study after the 2017 Subway Fire in Hong Kong, China

Emily Ying Yang Chan [1,2,3,4,*], Zhe Huang [1], Kevin Kei Ching Hung [1,4], Gloria Kwong Wai Chan [1], Holly Ching Yu Lam [1], Eugene Siu Kai Lo [1] and May Pui Shan Yeung [1]

1 Collaborating Centre for Oxford University and CUHK for Disaster and Medical Humanitarian Response (CCOUC), The Chinese University of Hong Kong, Hong Kong, China; huangzhe@cuhk.edu.hk (Z.H.); kevin.hung@cuhk.edu.hk (K.K.C.H.); gloria.chan@cuhk.edu.hk (G.K.W.C.); hollylam@cuhk.edu.hk (H.C.Y.L.); Euglsk@cuhk.edu.hk (E.S.K.L.); may.yeung@cuhk.edu.hk (M.P.S.Y.)

2 Nuffield Department of Medicine, University of Oxford, Oxford OX3 7BN, UK

3 François-Xavier Bagnoud Center for Health & Human Rights, Harvard University, Boston, MA 02138, USA

4 Accident and Emergency Medicine Academic Unit, The Chinese University of Hong Kong, Hong Kong, China

* Correspondence: emily.chan@cuhk.edu.hk;

Survey Questionnaire

1. What is your predominant mode of daily transport?

(a) Walk/cycle; (b) Subway; (c) Bus; (d) Private car; (e) Other modes.

2. Did you ever receive first aid training?

(a) Yes; (b) No.

(For question 3-9, please use 1-6 points to evaluate the following sentences regarding public transportation and disaster response. 1 point means to totally disagree, 6 points means to totally agree)

3. My daily transport is safe.

totally disagree 1 2 3 4 5 6 totally agree

4. I am worried that the MTR fire accident will occur on me.

totally disagree 1 2 3 4 5 6 totally agree

5. I am worried that the MTR fire accident will occur on my family or friends.

totally disagree 1 2 3 4 5 6 totally agree

6. I am worried that disaster/incident will occur on the daily transport I take

totally disagree 1 2 3 4 5 6 totally agree

7. I know how to deal with fire in transport

totally disagree 1 2 3 4 5 6 totally agree

8. I know how to deal with disaster/incident in community

totally disagree 1 2 3 4 5 6 totally agree

9. I am willing to learn more about community disaster preparedness

totally disagree 1 2 3 4 5 6 totally agree

10. Should water of room temperature or ice be used to the burn?

(a) ice water/ ice cube; (b) room temperature water.

11. If you are in a setting of fire incident and you found that someone was on fire, how would you use the fire blanket?

(a) put out the fire directly with the fire blanket; (b) cover the victim with the fire blanket and ask him/her to roll until the fire stop.

12. If there is no fire blanket at the scene, should fire hose or extinguishers be used on human?

(a) Yes; (b) No.
